# Clinical Holistic Medicine: Factors Influencing The Therapeutic Decision-Making. From Academic Knowledge to Emotional Intelligence and Spiritual “Crazy” Wisdom

**DOI:** 10.1100/tsw.2007.303

**Published:** 2007-12-10

**Authors:** Søren Ventegodt, Isack Kandel, Joav Merrick

**Affiliations:** ^1^ Quality of Life Research Center, Classensgade 11C, 1 sal, DK-2100 Copenhagen O, Denmark; ^2^ Research Clinic for Holistic Medicine, Copenhagen, Denmark; ^3^ Nordic School of Holistic Medicine, Copenhagen, Denmark; ^4^ Scandinavian Foundation for Holistic Medicine, Sandvika, Norway; ^5^ Interuniversity College, Graz, Austria; ^6^ Faculty of Social Sciences, Department of Behavioral Sciences, Ariel University Center of Samaria, Ariel, Israel; ^7^ National Institute of Child Health and Human Development, Jerusalem, Israel; ^8^ Office of the Medical Director, Division for Mental Retardation, Ministry of Social Affairs, Jerusalem, Israel; ^9^ Kentucky Children's Hospital, University of Kentucky, Lexington, KY, USA

**Keywords:** clinical holistic medicine, complementary and alternative medicine, decision making

## Abstract

Scientific holistic medicine is built on holistic medical theory, on therapeutic and ethical principles. The rationale is that the therapist can take the patient into a state of salutogenesis, or existential healing, using his skills and knowledge. But how ever much we want to make therapy a science it remains partly an art, and the more developed the therapist becomes, the more of his/her decisions will be based on intuition, feeling and even inspiration that is more based on love and human concern and other spiritual motivations than on mental reason and rationality in a simple sense of the word. The provocative and paradoxal medieval western concept of the “truth telling clown”, or the eastern concepts of “crazy wisdom” and “holy madness” seems highly relevant here. The problem is how we can ethically justify this kind of highly “irrational” therapeutic behavior in the rational setting of a medical institution. We argue here that holistic therapy has a very high success rate and is doing no harm to the patient, and encourage therapists, psychiatrists, psychologist and other academically trained “helpers” to constantly measure their own success-rate. This paper discusses many of the important factors that influence clinical holistic decision-making. Sexuality could, as many psychoanalysts from Freud to Reich and Searles have believed, be the most healing power that exists and also the most difficult for the mind to comprehend, and thus the most “crazy-wise” tool of therapy.

## INTRODUCTION

400,000 Danes used CAM (complementary and alternative medicine) in 1990, which is holistic and alternative medicine (defined as non-biomedical complementary, alternative, integrative or psychosocial interventions for medical purposes). This increased to 800,000 by year 2000[1] and is now expected to be 1,600,000 in 2010. If the development continues, as it has done in the United States already, there will be more CAM consultations than biomedical consultations in Denmark year 2020.

In spite of all this activity, the effect of CAM in general is still not clear at all. This is primarily because the term now refers to hundreds of treatment systems focusing on "the whole patient" and not primarily on symptoms or diseases, as is normal practice in today's mainstream biomedicine (pharmacological medicine).

What works in holistic medicine is healing of the patient's existence, called "salutogenesis" by Aaron Antonovsky (1923–1994)[2-9]. This is most often done by creating a deep shift in the consciousness of the patient towards a more positive and constructive attitude towards self, including body and mind, other people, and the world at large. The reason for the medical efficiency of such a shift towards positive attitudes and behaviors seems to be that consciousness is the primary determinant of global quality of life (QOL), health, and ability in general[10-14]. Because of the appreciation in the causal power of consciousness, many physicians and therapists are now focusing on this important shift in the patient's consciousness as their primary goal in treatment, when they want to improve QOL, health, and ability of the patient. This focus has caused the emerging field of scientific holistic medicine, i.e. "clinical holistic medicine"[15-55].

We have recently been able to document that such an approach can help every second patient in the patients own experience - with physical illness and chronic pain, mental illness, low self-esteem, sexual dysfunction, low quality of life, and low working ability[56-62]. Most interestingly we tested the holistic therapy on patients that could not be helped by their doctor with standard treatment (drugs), and many of the patients had had their chronic conditions for many years. This indicates quite a powerful effect of scientific holistic medicine. The clinical decision making were guided by many sound and rational theories and principles, but the different treatments took so many different routes that we literally invented a new cure for every new patient, leaving us with a need of deep reflections on what really is happening in the therapy. What are the "unpredictable" factors that are so radically influencing the therapists decisions, when not the rational principles of healing and therapy themselves? From where comes the surprising creativity in the session that in the end seemingly sets the patient free?

### The Therapeutic Principles and The Importance of Therapist's Fluency In Using Them

Since Hippocrates, holistic healing has been guided by medical principles[63]. The better the holistic therapist knows and understands these principles, and the more fluent he is in using them, the more efficient will the therapy be and the more lasting the results. Holistic therapy uses primarily four core principles of treatment[56]:

- Induce healing of the whole existence of the patient (salutogenesis) and not only his/her body or mind[2-9]. The healing often included goals like recovering purpose and meaning of life[64-72] by improving existential coherence[71] and ability to love, understand, and function sexually[67].

- Adding as many resources to the patient as possible as the primary reason for originally repressing the emotionally charged material was lack of resources — love, understanding, empathy, respect, care, acceptance, and acknowledgment — to mention a few of the many needs of the little child[17,49,68]. The principle was also to use the minimal intervention necessary by first using conversational therapy, then additional philosophical exercises if needed, then adding bodywork or, if needed, adding role play, group therapy, and finally when necessary in a few cases, referring to a psychiatrist for psychopharmacologic intervention[49]. If the patient was in somatic or psychiatric treatment already at the beginning of the therapy, this treatment was continued with support from the holistic therapist.

- Using the similarity principle[see 56 for references] that seems to be a fundamental principle for all holistic healing[63]. The similarity principle is based on the belief that what made the person sick originally will make the patient well again, when given in the right, therapeutic dose. This principle often leads to dramatic events in the therapy and to efficient and fast healing, but seems to send the patient into a number of developmental crises that must be handled professionally[50-52].

- Using Hering's Law of Cure[see 56 for references] to support the patient in going once again through all the disturbances and diseases, in reverse order, that brought the patient to where he or she is now. Other important axioms of Hering's Law of Cure is that the disease goes from more to less-important organs, goes from the inside out, and goes from upside down. The scientific rationale for the last three axioms are less clear than for the first: The patient must go back through his/her timeline in order to integrate all the states and experiences he/she has met on his/her way to disease. Going back in time is normally done though spontaneous regression in holistic existential therapy.

These four principles seem to be a lot to keep in mind, when you are practicing therapy, but you will soon learn that they are all aspects of the same fundamental principle, the abstract law of integration – everything to emotionally intense in your patients life must be felt again, recalled and understood, and finally "melted" into the patients own, natural understanding of life and being. This is the same as the patient returning to being him- or her self. So in this respect everything gets simpler as you get more experienced as therapist. Get your patient back into contact with the world through body and mind, heal the patients psychoform and the somatoform dissociation, just restore the patient's sense of coherence, and your are home free. But in practice therapy develops paradoxically more and more complex and more and more simple at the same time - more and more complex for the therapist's mind, and more and more simple for the therapist's self.

## What Is A Decision? Decisions Before and Decisions During Treatment

One rational way of understanding the development of a treatment of a patient is as a series of rational choices each one serving the purpose of using tools for healing the patient's life. This is a nice idea, and highly popular with academic thinkers. Unfortunately most choices in therapy are not based on ratio and reason, but on emotions, feelings, sensations and intuitions. This is because we are dealing with emotions. Therapy is about integrating difficult emotions. But still there is a consciousness and a will guiding these choices.

Philosophically, in the grand tradition of existentialistic thinkers, man has free will, and from that, free choice[73,74]. Choice is a consequence of the presentation in our human consciousness of more than one alternative, future action; the more conscious we are the more alternatives will be acknowledged by our self, and the wiser the choice, and thus the bigger the power and influence of the choice on our future destiny.

In the existentialistic philosophy of Søren Kirkegaard (1813-1855) we are divine beings empowered to create our own destiny good or bad. The empowerment comes from man containing in his innermost existential core the possibility to connect to the universe and from this connection in each situation draw the wisdom to make the good choice. When we loose this connection to the universe, we loose our existential orientation, and we fall into darkness and random choices, leaving responsibility for our live and relationship behind.

Sigmund Freud (1856-1939), Carl Gustav Jung (1875-1961) and their students elaborated on this further, defining the subconscious and the repression of emotions and sexuality, giving the science of psychodynamic therapy[75,76]. Antonovsky gave in the 1980s his theory of “sense of coherence”[2,3], which stated that the healthy person has a sense of coherence – inwards towards life and inner self making him alive, and outwards towards the world, making him real. Being alive and real is what a sound person is, and loss of health is loss of the sense of coherence making the person emotionally dead, mentally delusional, and spiritually aloof.

Resent developments in research on therapy have identified that this sense of coherence has two main vehicles, the mind and the body. The sense of coherence can be lost in part, when one of our to channels to the world shut down, either as somatoform or psychoform dissociation. Or both these vital channels can b closed leaving the patient without any real contact to the outer world, in a severely ill state, often suffering from both mental, existential, physical, and sexual problems and illnesses.

Rehabilitation of the connectedness to life and to the world, i.e. rehabilitation of the sense of coherence, is also the rehabilitation of the patient’s life, power, wisdom, and freedom of choice. This total healing of the patients existence, the existential salutogenesis, is the primary intention in scientific holistic therapy; this fundamental shift from not being into being see seems to be the central theme of the works of Kierkegaard on “hjaelpekunst” (Danish: the art of helping) and the focus of the old holistic medical tradition going all the way back to Hippocrates, who calling his noble medical art of helping and healing for “the art”[63]).

In practice the therapist will make many choices in each treatment, but as the fundamental problems of revitalization and existential rehabilitation in holistic therapy are pretty much the same with each patient, the choices seem to repeat themselves. The uninspired, experienced therapist will tend to take therapy into a boring and non-productive state of quite mechanical repetition, which is the dead of efficient therapy; when routine and dullness takes over the love dies; the investment of the therapist’s libidinous energy in the relationship with the patient is closed down; and what is left is the remote and formal relationship. The experienced, but inspired, therapist will move in the completely opposite direction, into a state of being where there is almost no choices left, but just a stream of consciousness and dancing libidinous energy, in witch the whole therapeutic setting and therapeutic process is embedded.

## The Therapist As The Tool

To be in flow [77], to be conscious [78], and to be happy (in the state of sat-shit-ananda: present, knowing and happy) seems to be the holistic therapeutic ideal of a human being; this can be further developed into non-knowing (cp. Zen: “state of no mind”), just being dancing with the patient’s consciousness in a state where all decisions are not made and the action never becomes a problem. This is the intuitive state of the experience holistic therapist, coming from love, and being completely in service of his patient.

Holistic therapy and the process of existential healing is unwrapping the personal history of the patient, sending him back to heal all wound on body, mind and soul, rehabilitating the “natural philosophy” of the patient, that is: the understanding of life that best serves his character and purpose of life. Unfortunately every therapeutic action is intensively impacting the philosophy of life of the patient, actually implanting philosophy in the patient; this philosophy must be de-learned for the natural philosophy of the patient to emerge.

The holistic therapeutic principles includes the most important principle of similarity; the patient will have to transfer his past into the present and transfer the emotional charges of his childhood traumas on the therapist With out this actualization of the past, the therapy cannot work, as already noticed by Freud. As we are transferring from both our bodily (emotional, sexual), and mental (philosophical, energetic) and spiritual (consciousness, love) realms, holistic therapy is often extremely complex, and much too complex to monitor by the therapist brain-mind. The body-mind (instinctive domains) and spirit-mind (intuitive domains) must be strongly involved for the therapist to be effective and successful as healer.

The most important thing in holistic therapy is the state and quality of the tool (one self as therapist), and personal development to a state where unconditional love to all human beings is natural is a sine qua non; unfortunately most therapist reaches this level late in life, if the therapist’s own therapy is not intensified and boddhahood actively pursued. Only in the state of unconditional love can the therapist be pure and unselfish and coming from his heart in service; only love allows the therapist to use all aspects of him self without hesitation to help the patient to heal his existence. Only another person's true love can set a tormented soul free, and that is what holistic healing is all about. And when you love, most choices are easy, because your personal interests are suspended, and all that matters is what best can help your patient. When the patient feels your love as a therapist, he or she will let go of the neurotic control that for survival reasons has replaces responsible conscious being, and the existence will heal and re-emerge.

## The Therapist's Philosophy Of Life

Holistic, existentially oriented, therapy is basically about re-interpreting life and through a more containing philosophy of life being able to integrate past events. The expansion of the patient's philosophy of life is done by consciously or unconsciously implanting a more accepting and loving philosophy of life in the patient. This is done through therapists body language, the way the clinic is decorated, the quality of therapist awareness, the concepts used, the attitude in the meeting, the nature of the therapeutic contract, the methods and technologies used, and of coarse primarily by the inspiration coming from meeting on a regular basis with a (hopefully) more sound and higher developed person than the patient him- or her self. Even if the relation is equal, holistic therapy can only work if the therapist is empowered by the patient by the patients illusion of the therapist being in some aspects wiser and superior – because most traumas and philosophical misunderstandings comes from the patients childhood, and the therapist must substitute the parent(s) to make the healing happen.

The therapist's own philosophy of life therefore becomes of crucial importance, and the more evolved and deep-sighted the therapist's philosophy is, the more efficient can he plant the containing philosophy. Truthfulness and honesty about the philosophical implantation will ease the process of de-learning the philosophy in the final stage of therapy. When the therapist is honest the patient will suffer, and this suffering is the patients meeting with reality, that in the end will restore sense of coherence with the outer world. The loving acknowledgment will support the patient in rehabilitating the sense of coherence inwards towards life and the deeper existential and spiritual layers (the "soul").

It is therefore helpful for the therapy that the therapist has values such as: honesty, openness, directness and compassion as a part of his actively worded philosophy.

## Therapist's Understanding Of The Therapeutic Process and Therapist Expectation

The processes of healing the existence is quite predictable and in an abstract sense always the same (see Figure 1). The movement is from the body (holding the repressed material) to the mind (denying responsibility by negative philosophy of life) to the spirit (the original cause of the problems by the historic unwise choices of the original, spiritually awake being). In the process of healing there are obligatory developmental crises, which the patient must go through to rehabilitate ability of love, understand and be in a sexual body. The better the therapist understand the process and the nature of the crisis, the better can the patients resistance and problems in the therapy be handled.

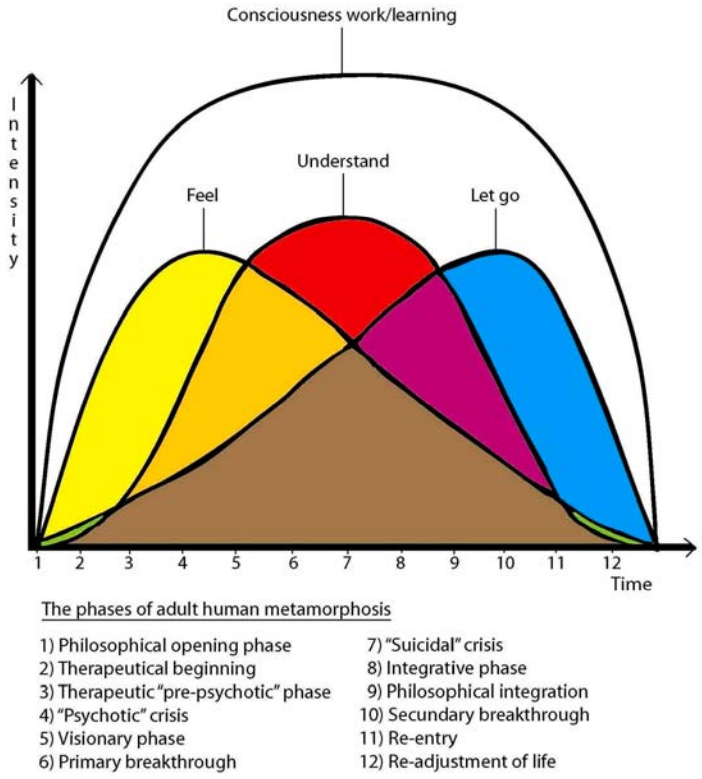

**Figure 1.** The process of holistic healing seen as three phases of feeling (yellow), understanding (red), and letting go (blue) of negative beliefs, attitudes, and decisions. As an end result, the process was improving the patient's philosophy of life and thus allowed the patient to rebalance existence and to assume responsibility for life. During the process, the patient's will re-established quality of life, health, and existential coherence, along with the ability to love, understand, and enjoy the whole spectrum of feelings and emotions, including sexuality.

Many patients in intensive therapy experience the healing as a series of phenomena or breakthroughs and existential crises with characteristic content. The most intense crises are metaphorically called the "psychotic", the "visionary", and the "suicidal" crises. They include feelings of going insane, not knowing the world or oneself, and wanting to die. Knowing what is coming next in the course of therapy is of great help to the patient, making it much easier to confront and integrate the often extremely intense, painful emotions and states of being, arising from integrating the early childhood traumas. The 12 steps (see Figure 1) are some possible steps in the process of healing and human transformation; understood though an ancient and powerful metaphor as the steps of "human metamorphosis"[52].

## The Therapists Understanding Of Health and Disease

Man consists of body, mind and spirit, and many of the energies are going though all aspects of the human being, like sexuality, meaning, and sense of coherence. The highly complex construction of the human being through billions of years of evolution and the limited ability to represent complexity in the brain-mind, the brain after all just being a small part of the human being, makes understanding health and disease one of the most central problems and most crucial issues in holistic therapy.

The modern holistic therapist must know a wide range of sciences from physics, biochemistry and biology, to medicine (anatomy, physiology, pathology), psychology, philosophy, and sociology. In the same time the therapist must be trained in art and literature, and he must also be deeply involved in the project of self-exploration, to develop a deep and thorough understanding of all aspects of self – from sexuality to spirituality.

The training and education of a holistic physician thus never ends. And many therapists get exhorted in the process of assimilating all existent scientific knowledge and ends up feeling insecure and insufficient. The temptation of closing ones view down to a specific therapeutic system with specific tools is big, but holistic medicine can newer work if the doctor himself is not the tool. The person cannot be substituted with procedures or machinery. And many therapists end up not working holistically, but just practicing some procedure and techniques mechanically, without the therapy healing existence and giving lasting effects.

## Hermeneutic Problems – Learning and De-Learning

The most fundamental problem of working professionally with induction of shifts in consciousness is the hermeneutic problems: that what we believe will be our reality. The reason for this is that our reality is a materialization of our consciousness[79]. Therefore we will always find confirmation for our beliefs in reality, in spite of our beliefs being in deep conflict with life itself, and with the larger world. This problem makes it a necessity for the holistic therapist to involve in a spiritual practice to develop consciousness.

Awareness of planting philosophy of life in the patient is a condition for de-learning the philosophy ion the end of the therapy. Not doing this leads to all the problems with dependence between therapist and patient, extended therapeutic courses with no progress lasting up to many years, and the famous problems of implanted memories, known from the trials where the family sue the therapist for implanting incest-memories – such “fake memories and ideas” are just the events of the patient’s personal history, interpreted though the “glasses” of the implanted philosophy, lasting after therapy because of lack of philosophical de-learning.

## Supervision and Therapist’s Personal Growth

The quality of the holistic therapist’s choices is because of hermeneutic problems completely dependent of second opinions; Balint group work and supervision is mandatory. The therapist must work in his own therapy with the existential problems that continue to be revealed because of a mirroring effect from the patients into the therapist – the famous process of counter-transference.

We have identified[71] nine key dimensions of existence, which exist in a passive and an active form, corresponding to the being and doing of life (see Table 1).

Interestingly, as a person develops, the nine areas merges completely; every part of existence becomes conscious, filled with love, meaningful, joyful, enlightened, purposeful, urge-driven, ecstatic and coherent, as all parts of existence expands into the neighbor areas. This expansion of all existential areas is the project of personal development, such as sex expands into the consciousness and love expands into sexuality we have the classical art of sexual tantra (see “the path of tantra”[71], that is integrating sex and consciousness). One by one all the splits and participations that torment modern man heal in this process of existential integration. Existential healing is therefore the primary goal of personal development[2,3].

**Table 1. The Nine Key Dimensions Of Existence**

| | Active form | Passive form |
|---|---|---|
| **1. Coherence, the web, the nest of the world** | Receiving, taking in | Being an integrated part |
| **2. Intent/purpose of life** | Intention, decision | Having a purpose (of life) |
| **3. Talent/strength** | Using skills and urges | Having strength and structure |
| **4. Consciousness** | Noticing, knowing, understanding, planning | Being awake |
| **5. Love** | Acting in love | Being in love |
| **6. Sex/physicality** | Meeting, enjoying | Being man/woman of character |
| **7. Light** | Bringing light | Being in light/enlightened |
| **8. Joy** | Bringing joy | Being in joy |
| **9. Meaning/QOL** | Creating/fulfilling life, giving | Being alive, having impact |

## The Therapist State Of Mind and State Of Being In The Therapeutic Process

The more relaxed, in flow, free, and happy, the therapist is, and the less he controls his rational and irrational impulses of talking and acting, the more flawless and efficient is the holistic therapy. Modern short-term therapy, where huge problems is intended solved in only 10 or 20 sessions, demands the therapist to be extremely active, in strong contrast to the old-style psychoanalytical therapist, who did almost nothing but listen to the patient while he did his free associations. Bodywork is becoming more and more common, and spiritual and philosophical exercises has become modern all over the western world as a part of holistic therapy. This puts new demands on the therapist to be ethically aware, and conscious about sexual transferences, emotional energies, symbiotic dependencies etc.

## The Therapeutic Tools - When To Use Them and When Not To Use Them

The ideal therapist uses only the loving and caring contact with the patient to induce holistic healing, the process Antonovsky called “salutogenesis”. But as we are not as loving as we potentially could be, our love is often not powerful enough to make the healing happen, and then we can go to using tools as a compensation for this lack of healing power. Unfortunately, tools are a meager substitute, and results obtained with tools are often temporary and not lasting long.

But as everything is a learning process, the acceptance of one limitations is an important prerequisite for growing, and daring to use tools to materialize ones firm intent of helping the patient to heal is the road to learn how to practice medicine, as it inevitably reveals our own impurities and shaddowish sides – if we dare to look, and we have someone to assist us by pointing at what we least of everything want to see in ourselves.

## The “Staircase” Of Therapeutic Tools Of Increasing Power[49]

As demonstrated throughout our many papers on clinical holistic medicine[15-55], almost everything can be used as a tool, since only the imagination sets the limit. To induce the state of consciousness we call “being in the process of healing”[17], the physician (according to Yalom[80]) needs to invent a new cure for every patient. This ability to be imaginative, creative, and use whatever is necessary to induce the healing is the hallmark of the excellent therapist. Good intent, balanced action, and good results are definitely needed in holistic medicine. Giving up on your patient and not doing anything at all might be a bigger sin, in many cases, than doing your best as a holistic physician and still losing your patient. Still you need to use any tool only after careful consideration, respecting the golden rule never to use a tool more powerful and dangerous than necessary (compare that both in surgery and with chemotherapy the patient is risking death as a result of the treatment).

Almost everything in the world can be used as a tool, but as the physician lines up his tools, some tools are used naturally before others and some might be painfully out of reach because of lack of expertise or due to the laws of your country. The ranking of tools after intensity, danger, and needed expertise of the physician gives a "staircase" of advanced tools of holistic medicine; its function is to help the holistic physician to "step up" in the use of the techniques one level at a time.

Let us admit that therapy often is a little "messy" with the combination of a number of tools and techniques. To think of therapy as the clear-cut process of "walking the staircase" is too simple. Often, many of the steps are used in subtle and symbolic ways by the skilled therapist, i.e., hidden in jokes and ironic remarks. So this staircase is meant for education, training, and treatment strategy, and not to limit the flexibility and spontaneity of the therapy.

The concept of "stepping up" in the therapy by using more and more "dramatic" methods to get access to repressed emotions and events has led to the common notion of a "therapeutic staircase" with still stronger, more efficient, and more potentially dangerous traumatic methods of therapy (see figure 1). We have identified 10 steps of this staircase:

- Is about establishing the relationship
- Is about establishing intimacy, trust, and confidentiality
- Is about giving support
- Is about taking the patient into the process of physical, emotional, and mental healing
- Is about social healing of being in the family
- Is about spiritual healing – returning to the abstract wholeness of the soul
- Is about healing the informational layer of the body (from old times called the ethereal layer)
- Is about healing the three fundamental dimensions of existence: love, power, and sexuality in a direct way
- Is mind-expanding and consciousness-transformative techniques, and
- Techniques transgressing the borders of the patient and therefore often traumatizing, like using force and going against the will of the patient.

When the holistic physician or therapist masters one step, he can go on to training and using the techniques of the next step of the staircase. As step 10 is often traumatizing for the patient even with the best of physicians, it is generally advised that the holistic physician or therapist do not go there. When mastered by the physician, steps 5–8 (9) can be used, when steps 1–4 do not help the patient sufficiently. The tools must be used one level at a time and each step implies an increasing risk for traumatizing the patient. Levels 8 and 9 often take many years of practice to master.

When everything else has been tried, but the healing has not occurred and the physician still senses that there is more to be done, the holistic physician can — if he has the necessary qualifications such as training in medical ethics and in the different treatment techniques, combined with a sufficient level of personal development and sufficient courage — use the advanced tools of holistic medicine. The advanced holistic physician's expanded toolbox contains powerful tools that can be organized into a staircase of the intensity of the therapeutic experience that they provoke and the level of expertise they take to master (see Figure 1 and Table 1). The more intense a therapeutic technique, the more emotional energy will normally be contained in the session and the higher the risk for the therapist to lose control or lose the patient to the dark side, which can make the therapeutic session very traumatic and damaging. These induced problems can almost always be healed if the patient stays in the therapy, so the real risk is losing the patient because he or she completely drops out of the therapy.

## Libidinous Investment In Abstinence As effective, Crazy-Wise, Therapeutic Behaviour

Most interestingly, the destiny of the therapist experience with therapy is his choice of closing down or opening up for his libidinous energy towards his clients; the most dangerous of these energetic openings are of cause the acceptance of the transference and counter transference of Oedipal love, because the temptations of not keeping the borders are biggest here. Harold F Searles stated in his brilliant paper "Oedipal love in the counter transference"[81] the thesis that it is the therapist's libidinous investment in sexual abstinence that helps mentally ill patients to recover; he is believed to have cured 40% of his schizophrenic patients by using the combination of a good heart and a brilliant administration of sexual energy to cure his patients. Using the therapists own sexuality in combination with a strict sexual ethic as the therapeutic tool is an example of a crazy-wise therapeutic behavior, that most people would abandon, if it was not for the fact that he cured so many patients and harmed no one. Most interestingly, if you are a firm believer in Freud's theory of libido as the only creative power of man, you will not find a libidinous investment in a patient "crazy-wise" or plain crazy, you would find it rational and well based on theory. From a crazy-wise perspective the Freudian concept of libido is a crazy-wise theory in itself.

## Intention and Spiritual Matters

The nature of the human wholeness is difficult to grasp as it is abstract; the essence of man – the essence of the soul – seems to be love in a particular color, the gift of the person, or the mission of the persons life[64-72]. When the patient recovers his remembrance of what he really is, the great talents of his personality are also revealed. Life is from this perspective about being of value to the world by using one's talents to enriching the surrounding world, and thus contributes in all relations.

The theory of existential coherence explains many of the same facets of existence covered by the Four Quadrant Theory of Ken Wilber[82]. He also started with "The Great Nest of Being", what we call the coherent matrix of energy and information, or the web of the world. Wilber's four quadrants are intentions, behavior, culture and social relations, but love is rejected as a central concept in Wilber's model, making this model less useful for deep holistic, existential therapy, where love, trust, and holding are prerequisites for taking the patient into the state of consciousness we call "being in the process of existential healing"[17]. Responsibility for the person's own world is also difficult to rehabilitate using the Wilber model, whereas this is the consequence of walking the path of responsibility, noticing and reacting to your own impacts.

## Research and Development Of The Science and The Therapist's Practice

Both human, culture, and society develops, and medicine must follow, if it is to be contemporary and helpful to modern man. But research is always about stepping over the borders of today and yesterday, and sometimes the decisions taken in a field of little experience will show wrong or insufficient.

In this field, making the wrong decisions is not only allowed, it is an obligation, for you cannot make any decision on incomplete foundation of knowledge without the attitude that it is completely OK to make mistakes, when you only learn from them and do whatever you can to make it up to the patient.

Research will naturally be done with the group of patients that cannot be helped with the standard method, and it is justified by their need for help. Often the case is that if the holistic therapist cannot help them, nobody can, as the biomedical doctor is sought first in most cases.

Both positive and negative results must be shared with the international community for the patients not to have suffered for nothing. The decision of doing something completely new in the intent of helping the patient on an experimental basis is the most difficult decision to make in the holistic clinic. Surprisingly, if the therapist remembers the principles of healing and make sure that the experimental treatment complies to these few basic rules, most new interventions will in our experience help the patient, also when all hope is lost. We have seen this with cancer patients, where chemotherapy has failed to help, and we have tried something untraditional to induce holistic healing; in most examples this has seemingly actually helped the patients to survive the life expectance given them by their biomedical doctor.

## Leaning From The Patient's Process and Self-Therapy

The attitude that "I as a physician" myself got a little of all diseases, imbalances, impurities, and disturbances is extremely helpful to accept the often dramatic impact on oneself from holistic therapy on the patient. The openness to learn takes the humility of a therapist who knows that he or she is not at all neither perfect nor completely sound. But to look deeply into your own wounds from being raised in a dysfunctional family with incestuous bindings are really challenging. And when it comes down to it, perfect parents are really rare. So we are all quite neurotic and damaged, and in need of healing our existence our self.

Helping other people knowing this about our self takes the challenge of being therapist to a new level. Surprisingly, the fact that therapy is provoking and inspiring our own personal growth, is what makes being a holistic therapist so satisfying and extraordinary. Only the painstaking process of personal growth will lead us to realize that there really are no limits for what we can do for our self and our patients.

## The Therapist's Healthy Attitude: Humility, Love and Acceptance

Coming from the heart is the solution to the problem of how to help. Because we are all caught in our mental description of the world, we will inevitably start our medical practice less holistic and more "methodological" and instrumental. But as we little by little realize that the drugs are not really helping much, and that other therapeutic tools and techniques are only excuses for intimacy, closeness and loving contact with the patient, we will day for day stand more bravely forward and finally admit, that we are beings of love, and that our natural tendency is to care and to give without getting anything but our own happiness in return. And in this realization we will grow into powerful holistic healers, in the same time, as we will feel more and more humble and powerless.

The paradox of love is that only when we let go, and accept that we really cannot do anything for another person, for the person must decide for himself, and create his own life – autonomously – for himself, can we help. This is the paradox and the miracle of holistic medicine. Being a successful therapist in this field is very much a question of surrendering to reality, being one with the Great Spirit, being purely of service, or how you want to put it.

## Patient's Metamorphosis, Miracle Cures, Religious Beliefs, and The Role Of The "Magic Healer"

The believe of most holistic healer is that the blue-print of body, mind, and spirit is always intact and that contacting this informational source within can lead to complete healing in spite of every seemingly misery and hopelessness of the situation. This is really a kind of religious belief, where life is in our imagination empowered with almost magic powers. Because of the logic of hermeneutics, this believe will often materialize, so the therapist that believe in true miracles will see them every day, and the therapist that don't will never see them. When the skeptical therapist enters the optimistic therapists clinic, he will find nothing but doubtful successes and certain failures, and when the trusting and positive therapist come to the skeptical physicians clinic he will find miracle after miracle happening even there.

Patients who come to believe will go to therapists that believe and here go though adult human metamorphosis[83-92] and be transformed into wonderful, able and happy people, even their bodies will be transformed. And patients that do not believe will go to therapist and get their bodies and minds damaged and destroyed. The religious healer will attract religious patients. And the skeptical healer will attract sceptical patients. Every person will get what he materializes. The therapist role is to serve and to materialise what he believes in. The holistic therapist will often believe in healing the whole existence. And all choices will be made in that believe.

## Relations To Biomedical Colleges, Other Therapists and Patient's Reality

The fact that biomedical and holistic therapist living in very different worlds with very different cosmologies and very different experiences is often becoming a problem for the patient, who has to chose between to fundamentally different worlds and different treatments.

And often are the skeptic minds much more powerful that the trusting souls, making biomedicine winning many legal and political battles. But all over the world people are more positive in their attitudes and philosophies, and holistic medicine is growing fast with more consultations now in the USA than biomedical consultations. The battles are becoming intensified all over the world, and it seems that we in the next 20 years or so will have a complete commercial shift into holistic medicine; this shift is already predicted and being prepared for by many of today's large pharmaceutical companies. In the same time we see increasing lobbyist activities from physicians and industry trying to suppress holistic medicine – the war against homeopathy in Germany being an example.

The war is happening in the way that the holistic medicine is tested on the premises of biomedicine. With homeopathy, it is most unlikely that it is the homeopathic drugs in themselves that has any effect; the healing happens as the patient becomes more conscious of his human character and thus more accepting and integrating in attitude and philosophy of life. But instead of looking of these shift in consciousness and acknowledging all the good things there is happening for the patients who believe, skeptical research is des-empowering the homeopathic tradition, obviously in the intension of substituting it with biomedicine ("rational medicine", "evidence based medicine").

There is really nothing evidence based about the way the war is going on; only materialization of believes, as both patients and researchers are caught in the hermeneutic illusory web of interpretation of the world. We need a truly integrative medicine now, with space for more that one cosmology, i.e. a poly-cosmological entrance. Because biomedicine is not wrong; from one perspective the world is really chemistry and physics only. And the spiritual medicine is not wrong either. From another perspective, everything is really a materialization of consciousness. It is time to embrace a poly-paradigmatic medical science.

Ethical consideration; monitoring and documenting the therapeutic results

The purpose of medical ethics is to ensure that the patient is not exploited or harmed in any way. To monitor the effect of the therapy and to be sure that it really helps and that it does not harm the patient is the primary ethical concern in holistic medicine. As the sense of coherence is the primary goal, and as this has been difficult to measure directly[4-9], the effect of holistic therapy on quality of life, health and ability has proven easy and efficient as an effect measure (it takes only 5 min. to fill in the QOL5 questionnaire[93] self-assessed QOL, subjective health (physical and mental) and the quality of human relationships); ability of functioning (love, work, social, sexual) is also relevant to measure.

To measure the patients before and after treatment seems to be mandatory, and we have done that for years in our clinic, being able to document sufficient results of the interventions on more than half the patients[56-61].

Bodywork is a hallmark of holistic therapies, and bodywork introduces a lot of ethical problems known already by Hippocrates and his students[63]. The ethical problems of modern bodywork might be best illuminated by using the extreme example of holistic sexological bodywork, originating both from the Hippocratic tradition and from the Asian/Indian holistic medical tradition[54,55]. The procedure of Hippocratic pelvic massage, in Denmark known and practiced by hundreds of therapists as "acupressure through the vagina" is such a technique that seemingly is extremely efficient to help patients with primary vulvodyni and chronic pelvic pain, but must be performed according to ethical standards. The holistic sexological procedures are derived from the holistic existential therapy, which involves re-parenting, massage and bodywork, conversational therapy, philosophical training, healing of existence during spontaneous regression to painful life events (gestalts) and close intimacy without any sexual involvement.

In psychology, psychiatry and existential psychotherapy[80], touch is often allowed, but a sufficient distance between therapist and client must always be kept, all clothes kept on and it is even recommended, that the first name is not taken into use to keep the relationship as formal and correct as possible. The reason for this distance is to create a safety zone that removes the danger of psychotherapy leading to sexual involvement. In the original Hippocratic medicine[63], as well as in modern holistic existential therapy such a safety zone was not possible, because of the simultaneous work with all dimensions of existence, from therapeutic touch[22] of the physical body, feelings and mind, to sexuality and spirituality. The fundamental rule has since Hippocrates been that the physician must control his behaviour, not to abuse his patient. The patients in holistic existential therapy and holistic sexology are often chronically ill, and their situation often pretty hopeless, as many of them have been dysfunctional and incurable for many years or they are suffering from conditions for which there are no efficient biomedical cure.

The primary purpose of the holistic existential therapy is to improve quality of life, secondary to improve health and ability. The severe conditions of the patients and the chronicity is what ethically justify the much more direct, intimate and intense method of holistic existential therapy, which integrates many different therapeutic elements and works on many levels of the patient's existence and personality at the same time. Holistic sexology is holistic existential therapy taken into the domain of sexology. The general ethical rule is that everything that does not harm and in the end will help the patient is allowed ("first do no harm"). An important aspect of the therapy is that the physician must be creative and in practice invent a new treatment for every patient, as Yalom has suggested[80]. To perform the sexological technique of acupressure through the vagina, the holistic sexologist must be able to control not only his/her behavior, but also his sexual excitement to avoid any danger of the therapeutic session turning into sexual activity. Most physicians can do the classic pelvic examination after their standard university training, but the vaginal acupressure we are discussing here in this paper can only be obtained through long training and supervision in order to reach a level, where such a procedure can be performed.

Side effects of the treatment can be soreness of the genitals and periods of bad mood, as old painful repressed material are slowly integrated. We have seen acute psychosis as a sexually abused woman confronted her most painful experiences, but she recovered in a few days without the use of drugs and this episode was an integral part of her healing. In fact it was her therapeutic breakthrough. As it is possible that the patient can feel abused from transferences, it is extremely important to address this openly to prevent this situation. We recommend that the patient is contacted or followed for 1-5 years, to prevent and handle any potential long-term negative effects of the treatment. In spite of these problems we have found the treatment with holistic existential therapy combined with the tool of vaginal acupressure to be very valuable for the patients[54,55].

## DISCUSSION

There are many factors influencing the therapist's choice of action in the therapeutic session. We have presented it as if the therapist had the power of deciding what is going on. The reason that every treatment of a patient takes its own route might very well be that every patient because of his or her basic resistance is struggling very hard not go get well, not to get cures, not to get into the state of salutogenesis. The reason for this is clear from a psychodynamic perspective: The defenses are created for survival difficult situations in the past, and the patent will unconsciously feel like dying if these situations reappear in consciousness.

So therapy is a dance, or a fight, or a play; a complex pattern is created like always when to forces are almost of same size and opposite each other, and creating a chaotic, highly dynamic middle zone of whorls and constant changes.

Our list of factors influencing therapy might be complete useless, if it is so simple that the patient subconsciously is doing whatever possible for destroying the therapy, and the therapist just is following along as well as possible. Because then the "individual cure for every patient" is nothing but the patients escape route before he or she is finally caught, and the destructive, neurotic or psychotic survival patterns busted for good.

The argument that the large creativity observed in clinical holistic therapy is coming from the therapist emotional and spiritual intelligence, might just be the therapist's narcissistic positive interpretation of what it is like to be almost completely out of control in the session. Maybe it is not a deeper and wiser layer of the therapist taking over, but just the patient unconsciously fighting for his or her survival, and therefore naturally investing more energy and efforts and therefore being smarter than us.

Many of the tools of the advanced holistic medical toolbox are inducing dramatic feelings in the patient, and it is an art to know when to use and when to avoid using a specific tool. The truth is that in spite of all the rational principles only the emotional intelligence can provide us with the wisdom of when to use a tool, because of the extreme complexity of the human consciousness. The central thing is therefore that the therapist at all times is aware of his intentions, and certain that he is in good intend towards his patients and acting in accordance with all professional and ethical principles. It might be almost impossible to control this from outside; because of this measuring the results of therapy and being sure of really helping his patients might in the end be the most ethical the therapist can do.

## CONCLUSION

Clinical holistic medicine is curing every second patient – in the patient's own experience – from physical illness and chronic pain, mental illness, low self-esteem, low quality of life, sexual dysfunction and low working ability[57-62]. But the therapy is not following any nice and reproducible pattern, in spite of 4 rather clear therapeutic principles and a well-defined tool-box[49]. On the contrary every treatment has its own course, and we say that we need to invent a new treatment for every patient. We are in this paper identifying many of the factors that seem to come into play guiding the therapist's decision-making in the session. We are suggesting that crazy-wise aspects of the therapist are responsible for the creativity that in the end will take the patient into existential healing (salutogenesis).

"Holy madness"[94] is a well-known concept from eastern spiritual teaching (as a Goggle-search will show), and seems to be a very appropriate expression for what is going on in the therapy, inside the therapist when he is fully engaged. Most interestingly "holy madness" is also a very accurate description of the state of consciousness called "holistic healing"[17]. And maybe all the chaos and creativity is not really delivered by the therapist but much more by the patient him- or her self; as therapists we like to flatter our self with the idea of being in control, creating the cure, and helping the patient making the idea of the patient being responsible less attractive (but never the less very likely).

We suggest that the therapist that allows himself to be existentially absorbed and engaged beyond the mind in the therapeutic process, and who is able to use all aspects of himself, body, mind and spirit included in the service of the patient, is much more successful in inducing existential healing in the patient, than the classic, rational, distant, mind-oriented, physician who uses only reductionistic, and scientific principles and tools for therapy.

Spiritual commitment and love is what we firmly believe heal the patients; only by letting go of the minds firm grip on reality can love find its natural and full expression in the therapy. Sexuality and libidinous interest is a natural part of this, and the investment of libidinous energy without acting out sexually has been suggested as the key to entering the universe of "crazy-wise healing".

Only by allowing the energy to dance within our self, and make the therapeutic decisions that we instinctively know are right to free our patient, and allow our self to speak and act completely without censorship, can we be as natural and powerful as we need to be, to overcome the resistance – the dark side of our self and the patient in combination - and induce Antonovsky salutogenesis[2,3] – the healing of the patient’s whole existence that will be followed by recovery of illness, improving of the patient’s abilities, and recovery of the patients global quality of life.

## ACKNOWLEDGEMENTS

The Danish Quality of Life Survey and the Quality of Life Research Center was 1991-2004 supported by grants from the 1991 Pharmacy Foundation, the Goodwill-fonden, the JL-Foundation, E. Danielsen and Wife's Foundation, Emmerick Meyer's Trust, the Frimodt-Heineken Foundation, the Hede Nielsen Family Foundation, Petrus Andersens Fond, Wholesaler C.P. Frederiksens Study Trust, Else & Mogens Wedell-Wedellsborg's Foundation and IMK Almene Fond. The research was approved by the Copenhagen Scientific Ethical Committee under number (KF)V.100.2123/91 and later correspondence.
